# Weighted Random Support Vector Machine Clusters Analysis of Resting-State fMRI in Mild Cognitive Impairment

**DOI:** 10.3389/fpsyt.2018.00340

**Published:** 2018-07-25

**Authors:** Xia-an Bi, Qian Xu, Xianhao Luo, Qi Sun, Zhigang Wang

**Affiliations:** ^1^College of Information Science and Engineering, Hunan Normal University, Changsha, China; ^2^College of Mathematics and Statistics, Hunan Normal University, Changsha, China

**Keywords:** mild cognitive impairment, weighted random support vector machine cluster, classification, abnormal brain areas, resting-state fMRI

## Abstract

The identification of abnormal cognitive decline at an early stage becomes an increasingly significant conundrum to physicians and is of major interest in the studies of mild cognitive impairment (MCI). Support vector machine (SVM) as a high-dimensional pattern classification technique is widely employed in neuroimaging research. However, the application of a single SVM classifier may be difficult to achieve the excellent classification performance because of the small-sample size and noise of imaging data. To address this issue, we propose a novel method of the weighted random support vector machine cluster (WRSVMC) in which multiple SVMs were built and different weights were given to corresponding SVMs with different classification performances. We evaluated our algorithm on resting state functional magnetic resonance imaging (RS-fMRI) data of 93 MCI patients and 105 healthy controls (HC) from the Alzheimer's Disease Neuroimaging Initiative (ADNI) cohort. The maximum accuracy given by the WRSVMC is 87.67%, demonstrating excellent diagnostic power. Furthermore, the most discriminative brain areas have been found out as follows: gyrus rectus (REC.L), precentral gyrus (PreCG.R), olfactory cortex (OLF.L), and middle occipital gyrus (MOG.R). These findings of the paper provide a new perspective for the clinical diagnosis of MCI.

## Introduction

Mild cognitive impairment (MCI) is a clinical entity which represents a state of slightly cognitive deficits for age and education, but does not markedly affect activities of daily life (1, 2). Studies show that healthy controls (HC) convert to Alzheimer's disease (AD) at an annual rate of 1–2% (3). Nevertheless, the rate of MCI patients who progress to AD is between 10 and 15% per year (4), implying that MCI may be a high-risk state for developing AD dementia. At present, there is no exact therapy which could completely stop or reverse the progression of AD (5). It is hence crucial to identify MCI patients and explore pathological changes in their brains, in order to offer timely treatment and slow down the transition from MCI to AD.

Neuroimaging techniques play increasingly important roles in the investigation of brain dysfunctions of MCI patients (6). In particular, the resting-state functional magnetic resonance imaging (RS-fMRI) may be one of the most popular brain imaging techniques due to its numerous advantages (7). On the one hand, RS-fMRI has higher spatial resolution than electroencephalogram (EEG) (8). On the other hand, RS-fMRI is noninvasive compared to position emission tomography (PET) and computed tomography (CT) (9). In addition, RS-fMRI is easier to implement without requiring specific tasks when compared to task-state fMRI (10). The application of RS-fMRI could help to enhance the understanding of spontaneous brain activities of MCI patients.

Graph theory is a reliable approach which offers a suitable framework for the study of brain neural network at a whole-brain connectivity level (11, 12). The literature on graph theory reports that MCI patients compared to HC show the altered functional connectivity (FC) and a clear disrupted topological pattern in the brain network (13, 14). Therefore, the graph theory metrics may possess predictive information that helps to classify MCI patients from HC. It is a promising approach that the discriminative graph theory metrics are considered as predictor features to build a classifier for excellent classification performance (15, 16).

Support vector machine (SVM) has been widely utilized for analysis of neuroimaging data to assist the identification of MCI (17). Zhang et al. (18) employed a linear SVM and achieved a classification accuracy of 79.02% for 346 MCI vs. 207 HC. Yu et al. (19) achieved an accuracy of 79.65% when using the SVM with leave-one-out cross validation to classify 170 patients with MCI from 169 HC. Zhang and Shen (20) reported an accuracy of 83.2% using the multi-modal SVM to discriminate between 91 MCI patients and 50 HC. Beheshti et al. (21) used the SVM classifier to yield a classification accuracy of 70.38% when distinguishing 87 MCI patients and 61 HC. Li et al. (22) achieved an accuracy of 77.4% for 99 MCI vs. 52 HC using the SVM. Because these MCI studies above usually considered a single SVM which may not be robust enough in dealing with neuroimaging data, the classification accuracies reported by the studies were universally lower than 85%.

To improve the accuracy and robustness of the classification algorithm, a novel approach of weighted random support vector machine cluster (WRSVMC) was put forward in this paper. Compared to a single SVM classifier, the WRSVMC has the following advantages: (1) The WRSVMC is robust because it consists of a great deal of SVM classifiers; (2) The classification accuracy of the WRSVMC is improved because the influences of strong SVM base classifiers are enhanced by a weighted method; (3) The abnormal brain areas could be found out using the WRSVMC based on the optimal subset of features; (4) The WRSVMC achieves an high accuracy of 87.67%, indicating that the abnormal brain areas which we have found were considerably convincing. In the process of exploring the abnormal brain areas, brain areas are ranked in accordance with the amount of discriminative information. We mainly discussed the first four brain areas as follows: gyrus rectus (REC.L), precentral gyrus (PreCG.R), olfactory cortex (OLF.L) and middle occipital gyrus (MOG.R). The gyrus rectus is considered to be a newly discovered abnormal brain area in patients with MCI because it is rarely studied in neuroimaging literature on MCI. The remaining three abnormal brain areas are consistent with the claims in existing literature involving MCI (23–25). In a word, these findings help us to understand the underlying pathologic mechanisms of MCI.

## Materials and methods

### Demographic information

The publicly available RS-fMRI data was obtained from Alzheimer's Disease Neuroimaging Initiative (ADNI) cohort (http://adni.loni.usc.edu/) (26) whose primary goal was to study the pathogenesis and treatment of MCI and AD by exploring multifarious imaging data (27). We initially collected 231 subjects' RS-fMRI data, including 93 MCI patients and 138 HC. 33 HC were excluded due to excessive head movements during the preprocessing, leaving 93 MCI patients (48 males and 45 females) and 105 HC (42 males and 63 females) for further analysis. We used Chi-squared test and found no significant discrepancy between the MCI patients and HC with respect to sex (χ^2^ = 2.683, *p* = 0.101). All data was anonymized according to the Health Insurance Portability and Accountability (HIPAA) guidelines, and followed the research procedures and ethical guidelines determined by the Institutional Review Boards (IRB) of the participating agencies.

### Data acquisition

All participants were imaged on a Siemens TRIO 3 Tesla machine. Resting state functional images were acquired using the scanning parameters as bellow: repetition time (TR) = 3, 000 ms, echo time (TE) = 30 ms, pixel spacing X/pixel spacing Y = 3.3/3.3 mm, acquisition matrix = 64 × 64, flip angle = 80, axial slices = 48, slice thickness = 3.313 mm, without slice gap, 140 time points. During the RS-fMRI scanning, all subjects should lie still and close their eyes without thinking of anything systematically.

### Data preprocessing

Image preprocessing was carried out by employing the Data Processing Assistant for Resting State fMRI (DPARSF) (www.restfmri.net) software. Briefly, the preprocessing steps were as bellow: converting data from DICOM to NIFTI format; discarding the first 10 volumes due to magnetization instability; correcting for time offset between slices; correcting for head motion between volumes; normalizing data with the echo-planar imaging (EPI) template; spatial smoothing using a Gaussian kernel with the full width-half maximum (FWHM) = 6 mm; linear de-trending; performing band-pass filtering (0.01–0.08 Hz); regressing out several spurious variables.

### The application of graph theory

Graph theory is an ideal approach to investigate the characteristics of the complex brain functional connectivity (FC) network. The application of graph theory is likely to help to improve the understanding of neural activities in the diseased and healthy human brain. In our experiment, we utilized the internationally common anatomical automatic labeling (AAL) atlases (28) to define the regions of interest (ROIs). Both the left and right brains could be divided into 45 ROIs, resulting in 90 ROIs. Each ROI is defined as a node in brain FC network. The time series of all voxels within each of ROIs are averaged to obtain the mean time series of each ROI, and the Pearson correlation coefficients are computed between each pair of mean time series. Therefore, a 90 × 90 FC network is constructed. Then a cut-off value in the range of [0, 1] is applied to FC network to get binary undirected graph. Specifically, the weight of the edge is 1 if there is an edge between two nodes, otherwise the weight is 0.

In this paper, the following graph theory metrics in the binary undirected graph are considered: degree, local efficiency, shortest path and clustering coefficient. These graph metrics are supported to be significantly different between the brain connectivity networks of HC and MCI patients (29–31). For each subject, 90 degrees, 4,005 shortest paths, 90 local efficiencies and 90 clustering coefficients are obtained and they are then utilized as classification features for subsequent experiments.

### The weighted random SVM cluster

#### The design of the WRSVMC

Machine learning techniques are widely utilized for pattern recognition, among which the SVM model shows excellent performance in classifying high-dimensional neuroimaging data (32). However, only a single SVM is not stable and there is a general problem of low classification accuracy for it. Bi et al. (33) proposed the random SVM cluster (RSVMC) which showed better generalization performance compared to a single SVM classifier. However, it is noteworthy that the performance of a single SVM classifier built in RSVMC may be considerably different. The RSVMC adopts a simple voting rule that the same weights are assigned to different SVMs, ignoring the differences between strong classifiers and weak classifiers. Therefore, there is still room for the improvement of the RSVMC algorithm.

**Figure 1:**
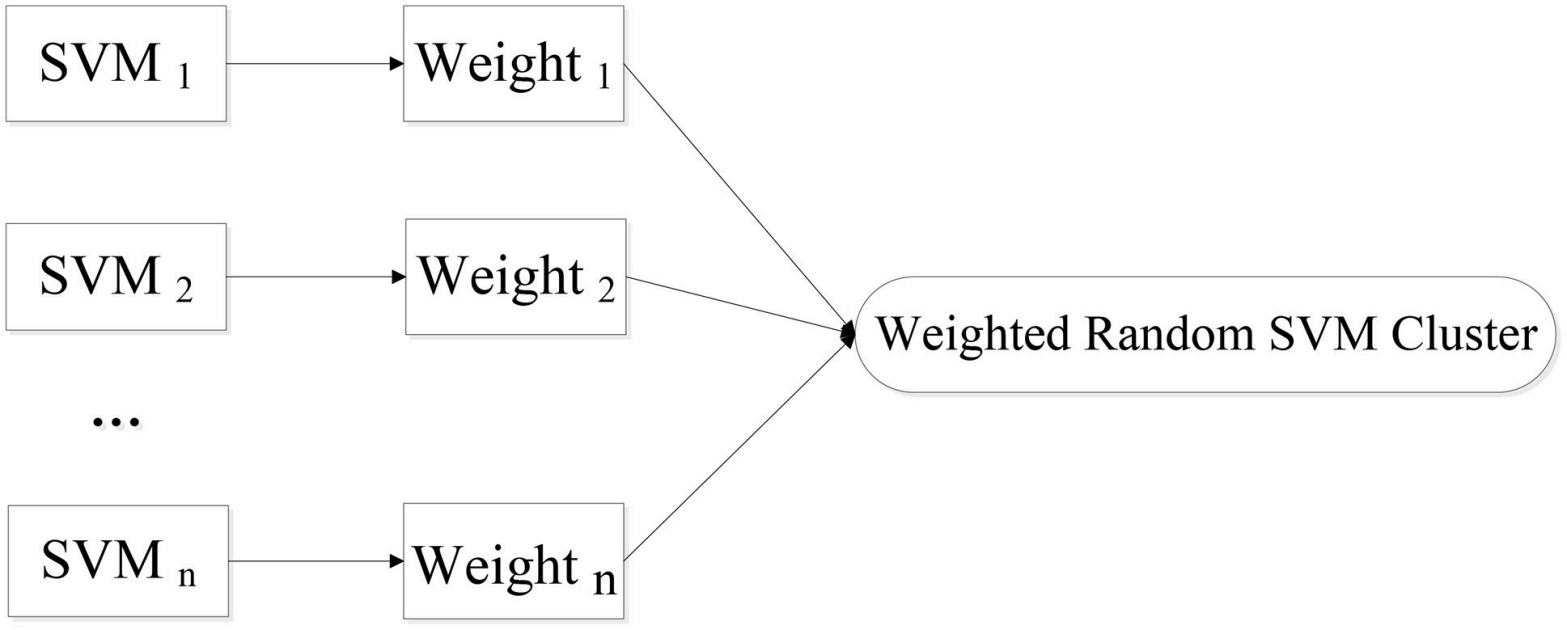
The idea of WRSVMC.

We put forward a novel approach of WRSVMC in this paper. Different weights are calculated for different SVM classifiers. The higher the SVM's accuracy is, the greater weight the SVM gets. As a result, the influences of the base classifiers with excellent classification performances are enhanced during the voting process, promoting the discriminative ability of the WRSVMC. Figure 1 exhibits the idea of our proposed WRSVMC.

**Figure 2:**
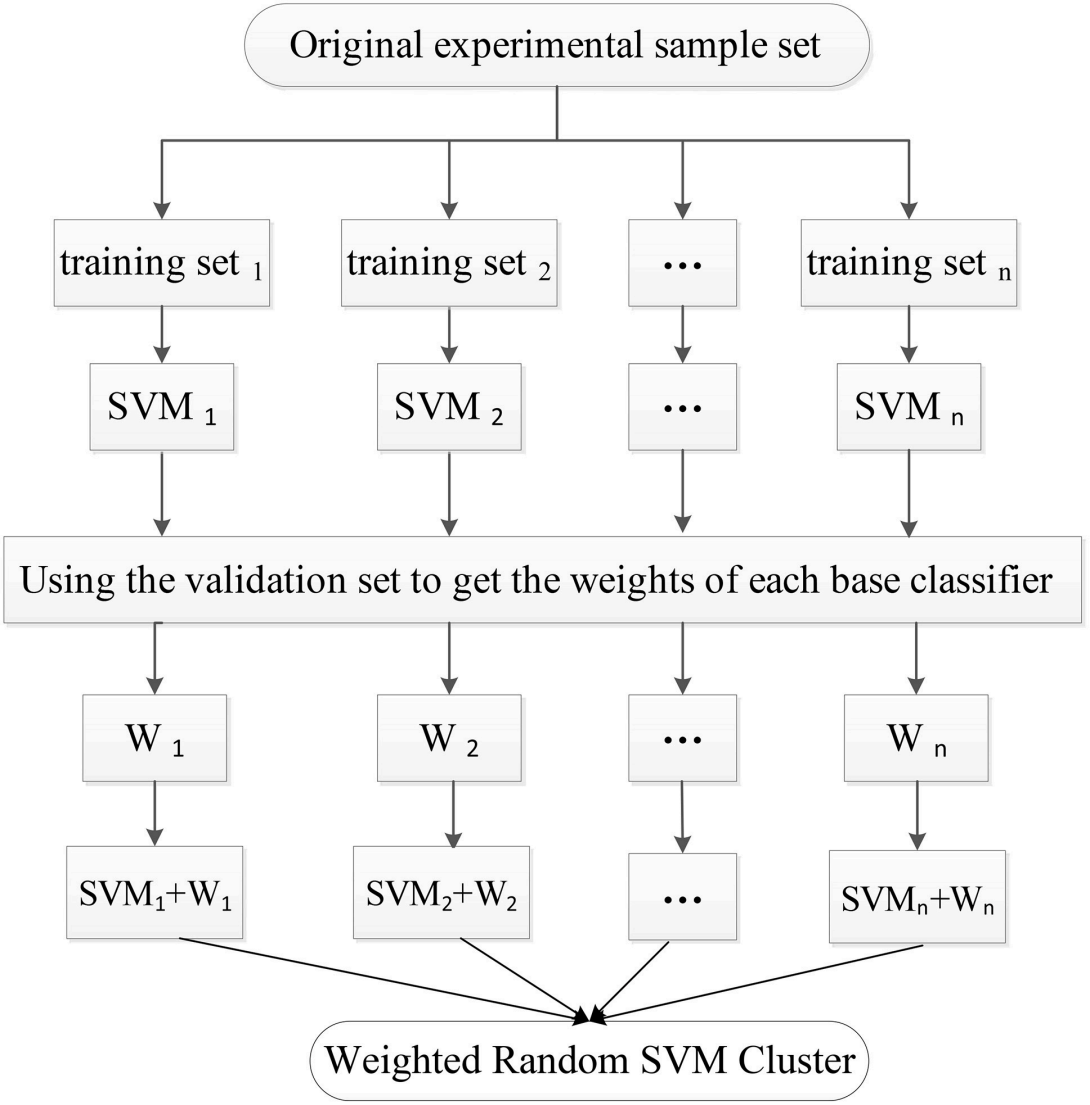
The training process of the WRSVMC.

In the first stage, the experimental sample set is split into the “training and validation” set and the test set. Then, the training process is followed. Specifically, a part of the samples are randomly selected from the “training and validation” set as the training set, and some features are randomly chosen from all features to construct a SVM classifier. The remaining validation set is used to calculate the single SVM's classification accuracy which is used as its weight. The training process is repeated for *n* times to obtain *n* SVM classifiers with weights, resulting in a WRSVMC which is more robust and accurate. Figure 2 describes the training process of the WRSVMC.

The WRSVMC could be used to forecast the class label of each test sample. Firstly, each test sample is fed into a WRSVMC classifier, and the amount of votes for each sample's label is weighted. The total amount of votes belonging to class *a* is denoted as *S*_*a*_

(1) $$\begin{array}{r} S_{a}=\sum_{i\;=\;1}^{n}\text{I}\left(f_{i}\left(x\right)=a\right)\times W_{i} \end{array}$$

where *x* represents a sample in the test set; *f*_*i*_(*x*) is the class label predicted by *i*-th SVM based on the test sample; (·) is the indicator function which takes values 0 and 1. If the test sample is predicted to be class *a*, the value equals to 1; otherwise, the value equals to 0.

Then the final predicted label *A* of the test sample is represented by the label with the maximum total amount of votes.

(2) $$\begin{array}{r} A=\text{Arg max}\left(S_{a}\right) \end{array}$$

By comparing the predicted label with the actual label, we could get the number of test samples that were correctly classified, denoted as *T*_*true*_. The classification accuracy *P*_*true*_ of the WRSVMC is given by:

(3) $$\begin{array}{r} P_{true}=\frac{T_{true}}{T} \end{array}$$

where *T* is the number of samples in the test set.

#### The classification of the WRSVMC

It is assumed that there are a total of *N* samples collected, of which *N*_1_ is the number of HC and *N*_2_ is that of MCI patients, where *N* = *N*_1_ + *N*_2_. Each sample has 4, 275 (90 + 4, 005 + 90 + 90) dimensional features, then marking the label of MCI patients as +1 and that of HC as −1. One of our tasks is to discriminate between MCI patients and HC based on 4,275 features.

First, 198 samples from ADNI cohort are split into 125 samples as the “training and validation” set and 73 samples as the test set. Next, 65 training samples are chosen from the “training and validation” set, and 65 features are picked out from 4,275 dimensional features to train a single SVM classifier. The cost parameter *c* for each SVM classifier is set to *Inf*, and the radial basis function (RBF) kernel is selected with a bandwidth σ of 3. Then, the remaining validation set is used to calculate the SVM's classification accuracy, which represents the weight of corresponding SVM. This training process is repeated for 500 times in the experiment.

The 73 test samples are put into the WRSVMC and each of 500 SVMs votes at the same time. The amount of votes for each SVM should be its weight, thus avoiding the disadvantages of voting with equal rights. The results of 500 SVMs are calculated and the class with the maximum total amount of votes is considered as the predicted class of the new sample. The number of the new samples that are correctly classified is divided by 73, which represents the classification performance of the WRSVMC.

The amount of base classifiers in the WRSVMC is initially set to 500. In general, with expansion of the amount of SVM classifiers, the WRSVMC could converge to lower generalization errors. But excessive SVM classifiers also increase experimental training time and even lead to overfitting. Therefore, the different amounts of SVM classifiers need to be tested. We use the classification accuracy of the WRSVMC as a guideline to decide the optimal amount of SVM classifiers in the WRSVMC.

#### Feature selection of the WRSVMC

Each SVM randomly selects features, resulting in different classification performances. However, the SVMs with high performances make more contributions to the performance of the WRSVMC. We extract the features of the above-mentioned SVMs and thus obtain the important features of the WRSVMC. Details are as follows.

Firstly, the 73 unseen samples are utilized to evaluate the performance of each of 500 weighted SVM classifiers. The SVMs with classification accuracies above 50% are considered to be effective classifiers and these SVMs would be retained in the WRSVMC. Then the value of each feature of the selected SVMs is multiplied by the corresponding weight as the score of the feature, denoted as *Score*_*i, j*_:

(4) $$\begin{array}{r} {\;Score}_{i,j}=\sum_{k\;=\;1}^{T}{\;H}_{k,j}\times W_{i} \end{array}$$

where *H*_*k, j*_ represents the *j*-th feature value of the *k*-th test sample.

**Figure 3:**
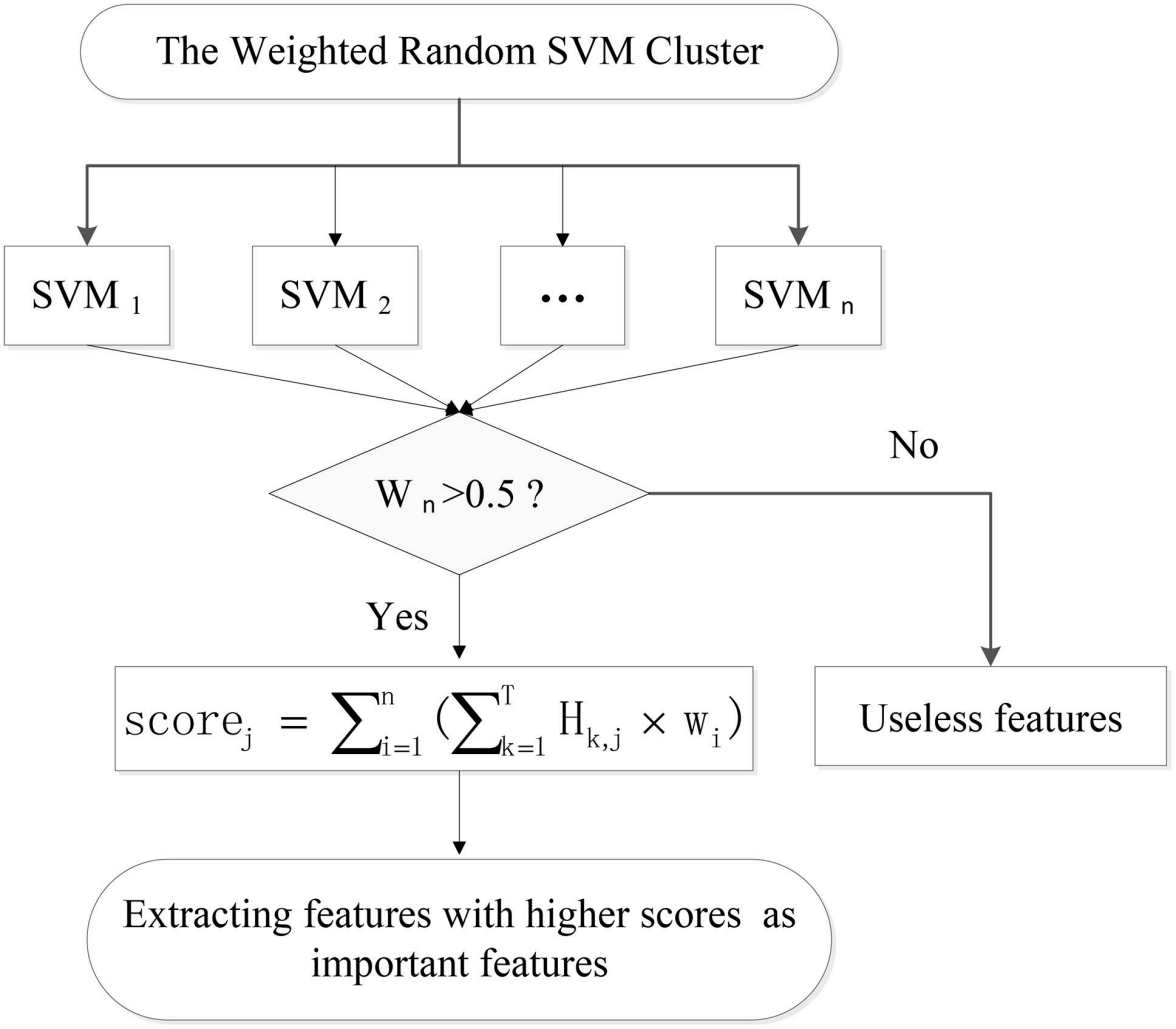
The extraction of important features.

The scores of the same feature are accumulated and the features ranking in the top 400 in terms of total scores are considered as important features (as shown in Figure 3).

(5) $$\begin{array}{r} {\text{Score}}_{j}=\sum_{i\;=\;1}^{n}{\text{Score}}_{i,j} \end{array}$$

Feature selection is conducted because of the fact that some input features are redundant and less relevant for the WRSVMC. Specifically, the 65 dimensional features are randomly chosen from the top *p* features of the 400 important features to perform the WRSVMC. We select a value for *p* from the set {70, 72, ⋯ , 400}. The classification performance of the WRSVMC is regarded as a guideline to find the optimal *p* in the experiment. The feature set of the top *p* features extracted from the 400 important features of the WRSVMC with the highest performance is considered as the optimal subset of features. As a result, the most discriminative features are chosen and meanwhile the redundant features are excluded.

In this study, we utilize the optimal subset of features to explore the most discriminative brain areas. Firstly, we detect the brain areas corresponding to each optimal feature. Then the brain areas are ranked in accordance with the frequencies of brain areas. The higher the frequency is, the more abnormal the brain area becomes.

## Results

### The performances of WRSVMC

**Figure 4:**
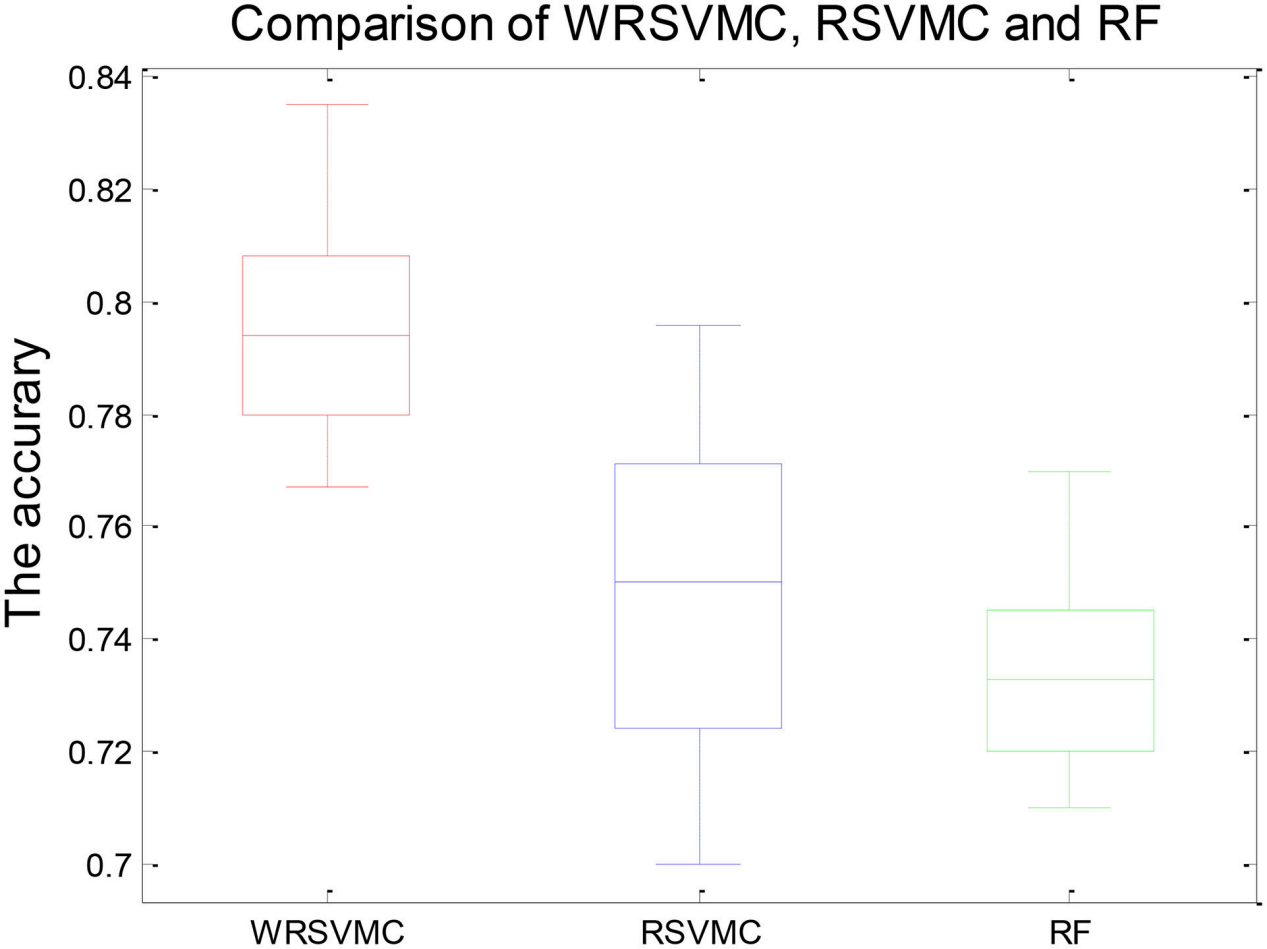
The generalization performance of the WRSVMC, RSVMC, and RF.

Figure 4 shows three boxplots comparing the generalization performances of the WRSVMC, RSVMC (33) and a non-SVM classifier, i.e., random forest (RF) which is an ensemble learner and has considerably wide applications in neuroimaging data. The box plots refer to the results of 50 experiments which perform these three classification algorithm respectively. It can be seen from the Figure 4 that the WRSVMC reports the comparatively higher classification accuracies in the range of 75–85% compared to the RSVMC with the range of 70–80% and the RF with the range of 70–78%. The maximum accuracy of the WRSVMC that we put forward is higher, and the overall performance is better.

Table 1 exhibits the statistical significance of results between the WRSVMC and other two methods. The two-sample *t*-test is conducted to examine the differences of the WRSVMC/RSVMC and WRSVMC/RF respectively and the *P*-values are close to 0.00 and 0.00, which indicates that the differences between the WRSVMC and other two classification methods are statistical significance. In addition, the complexities of these three ensemble classifiers depend on the number *n* of the base classifiers. Accordingly, all the complexities of the three algorithms are O(*n*). In a word, the experimental results show that our new WRSVMC is highly effective and stable.

**Table 1:** The statistical significance of results between the algorithms.

**Classifiers (Mean ± SD)**	**WRSVMC**	**RSVMC**	**RF**	***P-*value**
Accuracy (%)	0.80 ± 0.02	0.75 ± 0.03	0.73 ± 0.02	0.000^a^/0.000^b^

a *The P-value of the two-sample t-test between the WRSVMC and RSVMC*.

b *The P-value of the two-sample t-test between the WRSVMC and RF*.

### The optimal amount of base classifiers

**Figure 5:**
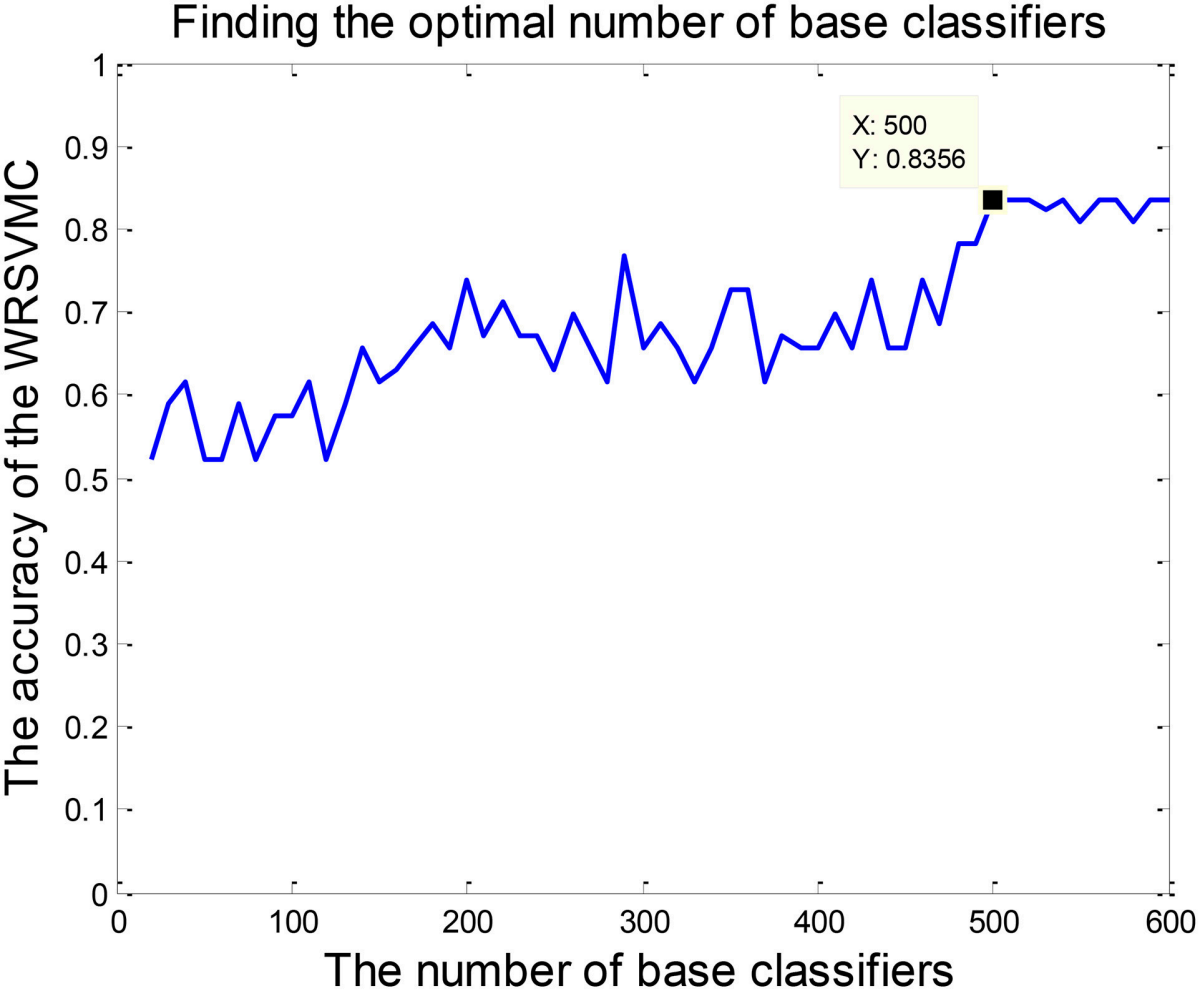
The optimal amount of SVM classifiers.

The amount of SVM classifiers in the WRSVMC with the minimum classification error is regarded as the optimal amount of SVM classifiers. In the first place, we gradually adjust the amount of SVMs from 20 to 600, with a step size of 10. Then, the classification performances of the WRSVMC with different amounts of SVMs are calculated. It can be seen from Figure 5 that our proposed WRSVMC based on all the original features achieves a maximum accuracy of 83.56%, and it becomes stable at the stage where the amount of the SVM classifier is 500. Therefore, 500 is selected to be the optimal amount of the SVM base classifiers.

### The important features

The important features should make important influences on the WRSVMC. We employ the score to measure the influence of each feature, and finally extract the features ranking in the top 400 in terms of scores as important features. Table 2 shows the features whose scores are rounded to 13, 12, and 11 sequentially. All of the features listed are the shortest paths between two ROIs, indicating that shortest path makes greater contribution to classification compared to other graph theory metrics. The features with the scores rounded to 13 or 12 are the shortest paths between ORBinf.L and IOG.L, IFGoperc.L and PCL.R, ORBinf.L and IOG.L, PHG.L and LING.L, PAL.L and MTG.L, HIP.L and PCL.R, SMG.R, and TPOsup.L respectively.

**Table 2:** The features with higher scores.

**Score (rounded)**	**Feature**
13	ORBinf.L-IOGL
12	IFGoperc.L-PCL.R PHG.L- LING.L PAL.L- MTG.L
	HIP.L -PCL.R SMG.R- TPOsup.L
11	REC.L- PHG.L SPG.R- MTG.R LING.L- HES.L SMG.L- ITG.L SMA.R- CAL.L ORBinf.L- ROL.R

### The optimal subset of features

**Figure 6:**
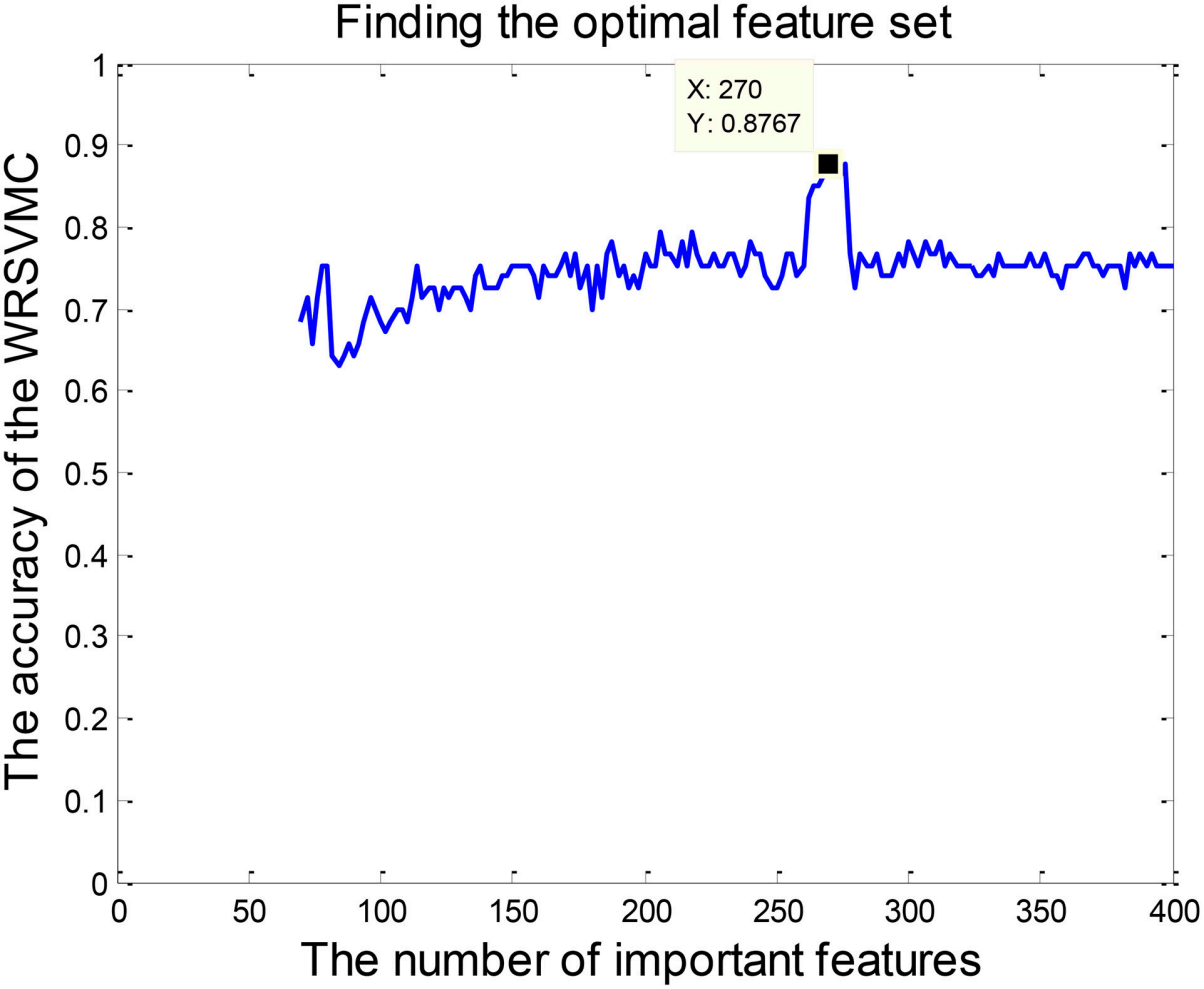
The number of optimal features.

Feature selection is performed for exploring the optimal subset of features from 400 “important features” to further enhance the final performance. The optimal *p* (70 ≤ *p* ≤ 400) could be found when the WRSVMC using the features set which consists of the top *p* features achieves the highest performance. As shown in Figure 6, the WRSVMC reports the highest accuracy of 87.67% when *p* is 270. Hence, the optimal subset of features comprises the top 270 dimensional features. At the same time, the WRSVMC achieves a sensitivity of 91.67% and specificity of 83.78% based on the most discriminative features. These features are used to explore the corresponding brain areas in the next experiment.

### The abnormal brain areas

**Figure 7:**
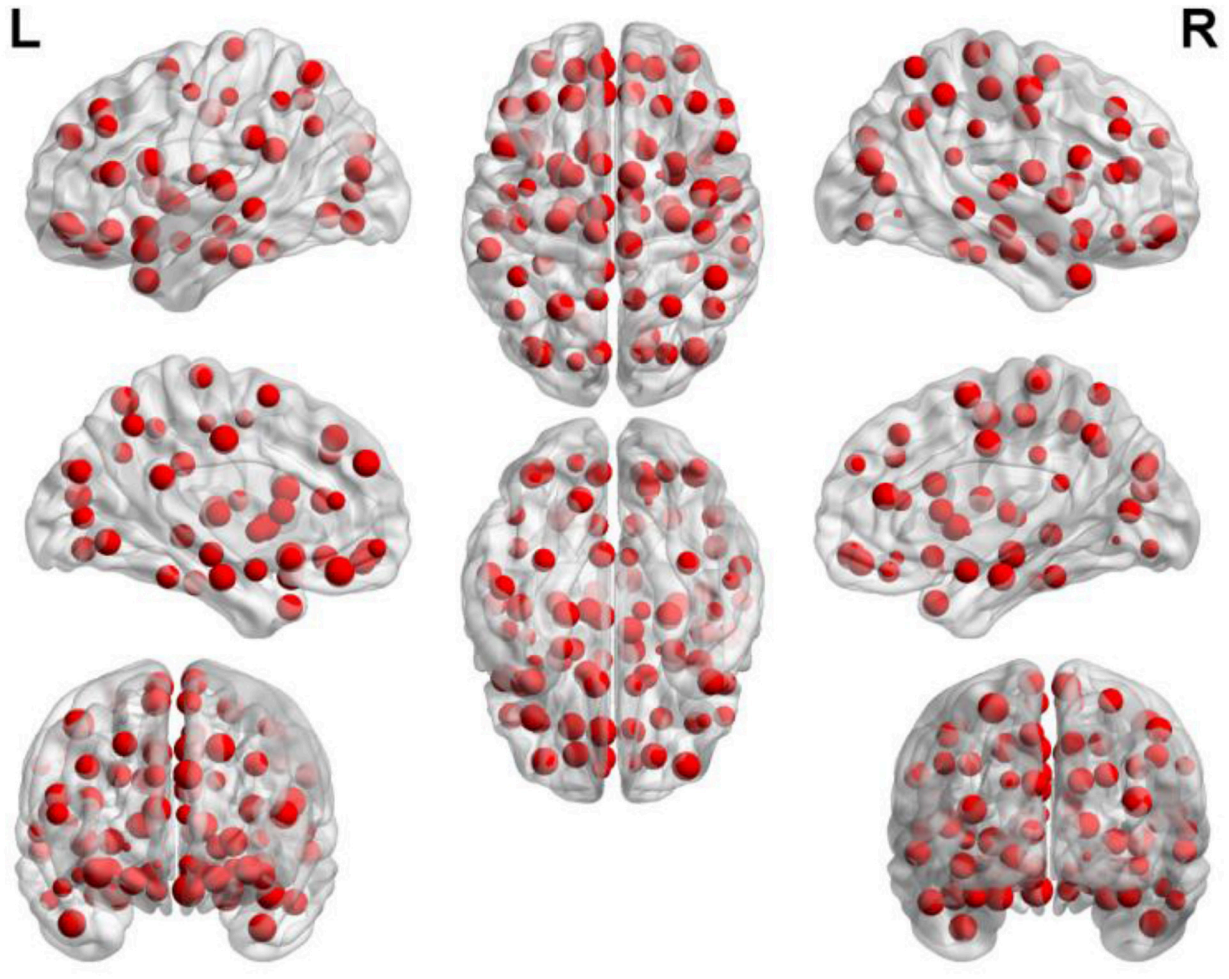
The frequency of each brain area.

Figure 7 depicts the locations of ROIs. Each node in the graph represents a ROI. The higher the frequency is, the larger the node becomes. The specific frequencies for some discriminative ROIs are shown in Table 3. The brain areas corresponding to the optimal subset of features with relatively higher frequecnies (11, 10 and 9) are as bellow: gyrus rectus (REC.L), precentral gyrus (PreCG.R), olfactory cortex (OLF.L), middle occipital gyrus (MOG.R), median cingulate and paracingulate gyri (DCG.L), superior parietal gyrus (SPG.L), inferior frontal gyrus (IFGoperc.L) and middle frontal gyrus (ORBmid.R).

**Table 3:** The brain areas with higher frequency.

**Frequency**	**Brain area**
11	REC.L
10	PreCG.R OLF.L
9	MOG.R DCG.L SPG.L IFGoperc.L ORBmid.R
8	SFGdor.L ORBmid.L SMA.R SFGmed.L INS.R ACG.R DCG.R HIP.R PHG.L PHG.R PCL.R PUT.L HES.L TPOsup.L

## Discussion

### Classification effect

In this paper, we combine RS-fMRI with the graph theory, and put forward a novel approach of WRSVMC to accurately distinguish MCI patients and HC. RS-fMRI is a reliable tool in mapping the brain FC networks due to its high-spatial resolution and noninvasive. Graph theory represents a powerful framework for the study of complex brain network properties. Furthermore, to the best of our knowledge, the WRSVMC is first applied to the neuroimaging data, which may be of great impact on neuroimaging research. The WRSVMC not only achieves a high accuracy of 87.67% (as shown in Figure 6), but also is employed to facilitate the detection of abnormal brain areas, which provides valuable insight into the diagnosis of MCI.

The SVM as a high-dimensional pattern classification technique has attracted more and more attention recently and has been showed to be an effective approach for the identification of MCI patients using medical imaging data. Zhang et al. (34) employed a multi-kernel SVM (MK-SVM) method for 91 MCI vs. 50 HC classifications and achieved an accuracy of 76.4%. Granziera et al. (35) used the SVM classifier to reach an accuracy of 75% when separating 42 MCI and 77 HC. Ye et al. (36) adopted the MK-SVM method fusing multi-modality data and achieved an accuracy of 82.13% discriminating between 52 HC and 99 MCI patients. Long et al. (37) reported an accuracy of 82.8% using the SVM based identification algorithm to classify 64 MCI patients from 60 HC. The performance metrics, e.g., accuracy, sensitivity and specificity of these SVM algorithms are listed in Table 4.

**Table 4:** The performance of our WRSVMC and existing SVM algorithm.

**Author**	**Method**	**Accuracy(%)**	**Sensitivity(%)**	**Specificity(%)**
(34)	MK-SVM	76.4	81.8	66
(35)	SVM	75	60	83
(36)	MK-SVM	82.13	87.68	71.54
(37)	SVM	83.1	82.8	83.3
This paper	WRSVMC	87.67	91.67	83.78

Most of the single SVM algorithms dealing with neuroimaging data possess the low classification accuracy because of the small number of samples and image noise. In addition, plenty of researches only focus on classification and rarely study abnormal brain regions associated with MCI. To address these problems, we innovatively propose the WRSVMC which represents the weighted ensemble of individual SVM, and produces better classification performance compared to a single SVM classifier. Feature selection is a crucial stage to deal with large-size feature vectors based on the graph theory metrics in our proposed WRSVMC algorithm. Specifically, we utilize the score to assess the influence of each input feature, and extract the top 400 features as the important features. Then, the classification performance of the WRSVMC is considered as a criterion to explore the optimal subset of features from the 400 important features. Finally, we utilize the optimal subset of features to search the corresponding brain areas which are mapped in Figure 7. The high accuracy of 87.67% (as shown in Figure 6) given by the WRSVMC suggests that the abnormal brain areas that we have found are considerably convincing.

In the process of building a WRSVMC, the training set is randomly selected from all the data and the features are randomly chosen from all the features, reflecting the randomness of the WRSVMC. As a result, each SVM classifier is considerably differentiated due to the random samples and random features, which could ensure that there is no overfitting issue during the training procedure of the proposed WRSVMC method to some extent. In addition, the WRSVMC works well on the test set, which demonstrates an excellent generalization performance, implying a very low possibility of overfitting.

In the experiment, some initial parameter values are set to build the WRSVMC. We now discuss whether these parameter values are appropriate. On the one hand, the cost parameter *c* for each SVM is set to *Inf* and the RBF kernel with a bandwidth σ of 3 is chosen. Although we artificially select these specific parameter values, we test other parameter values and find no considerable differences with respect to the classification accuracy of the WRSVMC, which indicates that the WRSVMC is stable and universal. On the other, a cut-off value of 0.25 is employed for the brain FC network. When a larger cut-off value is given, the network turns into more granular and fragmented. We conduct a grid search of different cut-off values and find that the optimal cut-off value is still 0.25.

### Analysis of the brain areas with higher frequencies

Our findings suggest that abnormal brain areas associated with MCI mainly involve in gyrus rectus, olfactory cortex, precentral gyrus, and middle occipital gyrus. Next, detailed analysis of these brain areas was discussed.

#### Gyrus rectus

The gyrus rectus possesses the highest frequency compared to other ROIs, which indicates that the gyrus rectus makes a great contribution to our WRSVMC algorithm.

The gyrus rectus is located in the frontal lobe's basal surface (38). The frontal lobe plays an important part in executive function, memory, decision-making and so on Fang et al. (39). Hence, the gyrus rectus may associate with cognitive and memory functions. Joo et al. (40) reported that the gyrus rectus resection had a temporary negative influence on memory recall and language. Qiu et al. (41) showed that the gyrus rectus played a vital role in efficient communications. Kristine et al. (42) found that the gyrus rectus was crucial to inhibit improper behavior. Georgiopoulos et al. (43) reported that the gyrus rectus may be relevant to executive function.

A great deal of previous literature showed that Alzheimer's disease (AD) was linked to abnormal gyrus rectus (44, 45). However, little was known about the relationship between MCI and abnormal gyrus rectus. Neuroimaging literature has shown that MCI is a precursor to AD (46, 47), indicating that MCI patients may have the certain gyrus rectus abnormality which is found in patients with AD. In this paper, we considered gurus rectus to be a newly discovered abnormal brain area in patients with MCI due to the highest frequency. Bahar-Fuchs et al. (48) found out considerable amyloid-β burden in gyrus rectus region of amnestic MCI patients compared to HC, which supported our findings to some extents.

The abnormal gyrus rectus is likely to bring about deficits in executive and cognitive functions and lead to memory loss in patients with MCI. The discovery of this new abnormal brain area provides a new perspective for the clinical diagnosis and intervention of MCI.

#### Olfactory cortex

The olfactory cortex obtains a relatively high frequency which suggests that the olfactory cortex plays a decisive role in our WRSVMC method.

The olfactory cortex refers to the classical cellular structure of nervous cortex, which is primarily involved in associative learning and memory (49). Yaniv et al. (50) observed the changes in the olfactory (piriform) cortex in the odor memory task. Daniels et al. (51) found out the crucial role of the olfactory cortex in emotional memory processing. Stone et al. (52) reported that the stimulation of olfactory cortex led to enhanced spatial memory. Goto et al. (53) showed that the verbal memory function had a positive correlation with the olfactory cortex volume.

The abnormal olfactory cortex was observed in numerous MCI studies. Zhang et al. (54) found out the disrupted connectivity between the right olfactory cortex and other hub areas in patients with amnestic MCI. Kirova et al. (55) mentioned that MCI patients showed neurofibrillary tau tangles and amyloid plaques in the olfactory cortex. Risacher et al. (24) found that olfactory cortex's *in vivo* activation was lessened in MCI patients. Vasavada et al. (56) observed the alterations of olfactory cortex activity in patients with MCI. Guzman et al. (57) discovered that olfactory cortex and hippocampus volume play the important roles in affecting memory impairment in patients with amnestic MCI.

The abnormal olfactory cortex may refer to a decline in higher-order memory processing and spatial cognitive function in MCI patients. The discovery of olfactory cortex provides assistance for clinical diagnosis of MCI.

#### Precentral gyrus

The precentral gyrus gets a comparatively high frequency which make clears that the precentral gyrus is a crucial part in our WRSVMC algorithm.

The precentral gyrus is a major motor cortex which is parallel to the central sulcus. Qiu et al. (41) mentioned that the precentral gyrus is involved in language, memory and motor functions, and have a large impact on efficient communications. Sakurai et al. (58) discoverd that the damage of the precentral gyrus led to acalculia with decreased verbal short-term memory. Sakreida et al. (59) found that the core regions of the precentral gyrus were activated when understanding the language content. Chang (60) reported that the 6-year-old children learning instrumental musical for more than a year showed alterations in the precentral gyrus.

Several studies of MCI reported the correlations between MCI and abnormal precentral gyrus. Chirles et al. (23) found that bilateral precentral gyrus showed increased correlations with other brain areas after an exercise intervention in the MCI group. Han et al. (61) pointed out significantly increased connectivity between the posterior cingulate cortex and the precentral gyrus in MCI patients. Rose et al. (62) observed considerably increased mean diffusivity measurements in the right precentral gyrus in MCI patients. Lin et al. (63) mentioned that the right precentral gyrus was identified with significantly interaction effects by employing the analysis of covariance in patients with MCI.

The abnormal precentral gyrus may lead to challenges in learning knowledge, sluggish behavior, and reduced executive functions in MCI patients. The discovery of precentral gyrus provides new insights into the identification of MCI.

#### Middle occipital gyrus

The middle occipital gyrus gains the relatively high frequency which means that the middle occipital gyrus has a significant influence on our WRSVMC method.

The middle occipital gyrus is the largest gyrus in the occipital lobe, which is the visual processing center of brain. Mickley Steinmetz et al. (64) observed that the amygdala activation was associated with modulation of the middle occipital gyrus when processing emotional stimuli. van Dam et al. (65) reported that the fractional amplitude of low frequency fluctuations (fALFF) had a significant correlation with short-term memory within left middle occipital gyrus. Lauer et al. (66) found that patients with abnormal middle occipital gurus had a poor performance on visual memory. Arsalidou et al. (67) found that static faces showed less activity than dynamic faces in left middle occipital gyrus.

The middle occipital gyrus abnormality was found in a mass of MCI studies. Jacobs et al. (68) found out the significantly increased connectivity from the right middle occipital/angular gyrus to the inferior parietal lobule in amnestic MCI patients. Alexopoulos et al. (69) observed the lower perfusion in the left middle occipital lobe in MCI patients. Makizako et al. (70) found that poor performance in the 6-min walking distance (6MWD) was linked to the decreased cerebral gray matter volume in middle occipital gyrus in MCI patients. Wang et al. (25) found out the significantly decreased FC between the middle occipital lobe and the left thalamus in MCI patients.

The abnormal middle occipital gyrus may result in visual memory impairment and cognitive loss in MCI patients. The discovery of middle occipital gyrus offers assistance for clinical diagnosis and discrimination of MCI.

## Limitations

The current study still has some limitations. First of all, the internationally accepted AAL template is employed to define the brain areas and the whole brain is divided into 90 ROIs, which leads to the fact that the division scale of the complex brain is still not small enough. The template for dividing the brain can be selected at a smaller scale to offer more informative and precise description for brain neural network. Then, the choice of graph theory metrics in this paper is based on the existing literature. However, there is no unified conclusion on how to select the most discriminative input features. With the deepening of research, more significant and meaningful predictor features can be considered to build the WRSVMC algorithm. Finally, the neuroimaging data we have obtained is the fMRI data of all subjects. Other modality of data could be utilized at the same time such as structural magnetic resonance imaging (sMRI), which could provide complementary information.

## Ethics statement

This study was carried out in accordance with the recommendations of National Institute of Aging-Alzheimer's Association (NIA-AA) workgroup guidelines, Institutional Review Board (IRB). The study was approved by Institutional Review Board (IRB) of each participating site, including the Banner Alzheimer's Institute, and was conducted in accordance with Federal Regulations, the Internal Conference on Harmonization (ICH), and Good Clinical Practices (GCP).

## Author contributions

XB proposed the design of the work and revised it critically for important intellectual content. QX and QS carried out the experiment for the work and drafted part of the work. XL and ZW collected, interpreted the data and drafted part of the work. All the authors approved the final version to be published and agreed to be accountable for all aspects of the work in ensuring that questions related to the accuracy or integrity of any part of the work are appropriately investigated and resolved.

### Conflict of interest statement

The authors declare that the research was conducted in the absence of any commercial or financial relationships that could be construed as a potential conflict of interest.

## References

[B1] Andrei C Manuela P Alin C Daniel T Cristinel S Ziad N General issues encountered while diagnosing mild cognitive impairment in Romanian patients. Int J Geriatr Psychiatry (2017) 32:116–7. 10.1002/gps.4531 27925375

[B2] Hampstead BM Sathian K Bikson M Stringer AY. Combined mnemonic strategy training and high-definition transcranial direct current stimulation for memory deficits in mild cognitive impairment. Alzheimers Dement Transl Res Clin Interv. (2017) 3:459–70. 10.1016/j.trci.2017.04.008 29067352 PMC5651427

[B3] Liu D Zhang L Li Z Zhang X Wu Y Yang H. Thinner changes of the retinal nerve fiber layer in patients with mild cognitive impairment and Alzheimer's disease. BMC Neurol. (2015) 15:14. 10.1186/s12883-015-0268-6 25886372 PMC4342899

[B4] Ramírez J Górriz JM Ortiz A Martínez-Murcia FJ Segovia F Salas-Gonzalez D. Ensemble of random forests one vs. rest classifiers for MCI and AD prediction using ANOVA cortical and subcortical feature selection and partial least squares. J Neurosci Methods (2018) 302:47–57. 10.1016/j.jneumeth.2017.12.005 29242123

[B5] Kunneman M Smets EMA Bouwman FH Schoonenboom NSM Zwan MD Pel-Littel R. Clinicians' views on conversations and shared decision making in diagnostic testing for Alzheimer's disease: the ABIDE project. Alzheimers Dement Transl Res Clin Interv. (2017) 3:305–13. 10.1016/j.trci.2017.03.009 29067337 PMC5651435

[B6] Gruber O F142. The use of neuroimaging markers in stratified diagnosis and therapy of schizophrenic and affective disorders. Schizophr Bull. (2018) 44:S275–5. 10.1093/schbul/sby017.673

[B7] Caceda R Bush K James GA Stowe Z Knight B Kilts C 471. Resting brain connectivity differentiates suicidal ideation from acute suicidal behavior. Biol Psychiatry (2017) 81:S192 10.1016/j.biopsych.2017.02.955

[B8] Turner J Damaraju E Van Erp T Mathalon D Ford J Voyvodic J. A multi-site resting state fMRI study on the amplitude of low frequency fluctuations in schizophrenia. Front Neurosci. (2013) 7:137. 10.3389/fnins.2013.00137 23964193 PMC3737471

[B9] Sechi G Demurtas R Boadu W Ortu E. Letter re: alterations of functional connectivity of the motor cortex in Fabry disease: an RS-fMRI study. Neurology (2017) 89:1842. 10.1212/WNL.0000000000004566 29061676

[B10] Wang J-B Zheng L-J Cao Q-J Wang Y-F Sun L Zang Y-F. Inconsistency in abnormal brain activity across cohorts of ADHD-200 in children with attention deficit hyperactivity disorder. Front Neurosci. (2017) 11:320. 10.3389/fnins.2017.00320 28634439 PMC5459906

[B11] Keown CL Datko MC Chen CP Maximo JO Jahedi A Müller R-A. Network organization is globally atypical in autism: a graph theory study of intrinsic functional connectivity. Biol Psychiatry Cogn Neurosci Neuroimag. (2017) 2:66–75. 10.1016/j.bpsc.2016.07.008 28944305 PMC5607014

[B12] Vecchio F Miraglia F Piludu F Granata G Romanello R Caulo M. “Small World” architecture in brain connectivity and hippocampal volume in Alzheimer's disease: a study via graph theory from EEG data. Brain Imag Behav. (2017) 11:473–85. 10.1007/s11682-016-9528-3 26960946

[B13] López ME Engels MMA van Straaten ECW Bajo R Delgado ML Scheltens P. MEG beamformer-based reconstructions of functional networks in mild cognitive impairment. Front Aging Neurosci. (2017) 9:107. 10.3389/fnagi.2017.00107 28487647 PMC5403893

[B14] Song J Birn RM Boly M Meier TB Nair VA Meyerand ME. Age-related reorganizational changes in modularity and functional connectivity of human brain networks. Brain Connect. (2014) 4:662–76. 10.1089/brain.2014.0286 25183440 PMC4238253

[B15] Hojjati SH Ebrahimzadeh A Khazaee A Babajani-Feremi A Alzheimer's Disease Neuroimaging I. Predicting conversion from MCI to AD using resting-state fMRI, graph theoretical approach and SVM. J Neurosci Methods (2017) 282:69–80. 10.1016/j.jneumeth.2017.03.006 28286064

[B16] Khazaee A Ebrahimzadeh A Babajani-Feremi A Alzheimer's Disease Neuroimaging I. Classification of patients with MCI and AD from healthy controls using directed graph measures of resting-state fMRI. Behav Brain Res. (2017) 322:339–50. 10.1016/j.bbr.2016.06.043 27345822

[B17] Asgari M Kaye J Dodge H. Predicting mild cognitive impairment from spontaneous spoken utterances. Alzheimers Dement Transl Res Clin Interv. (2017) 3:219–28. 10.1016/j.trci.2017.01.006 29067328 PMC5651423

[B18] Zhang J Liu M Le A Gao Y Shen D. Alzheimer's disease diagnosis using landmark-based features from longitudinal structural MR images. IEEE J Biomed Health Inform. (2017) 21:1607–16. 10.1109/JBHI.2017.2704614 28534798 PMC5685894

[B19] Yu K Wang X Li Q Zhang X Li X Li S. Individual morphological brain network construction based on multivariate euclidean distances between brain regions. Front Hum Neurosci. (2018) 12:204. 10.3389/fnhum.2018.00204 29887798 PMC5981802

[B20] Zhang D Shen D. Multi-modal multi-task learning for joint prediction of multiple regression and classification variables in Alzheimer's disease. NeuroImage (2012) 59:895–907. 10.1016/j.neuroimage.2011.09.069 21992749 PMC3230721

[B21] Beheshti I Maikusa N Daneshmand M Matsuda H Demirel H Anbarjafari G Japanese-Alzheimer's Disease Neuroimaging I. Classification of Alzheimer's disease and prediction of mild cognitive impairment conversion using histogram-based analysis of patient-specific anatomical brain connectivity networks. J Alzheimers Dis. (2017) 60:295–304. 10.3233/JAD-161080 28800325

[B22] Li F Tran L Thung KH Ji S Shen D Li J. A robust deep model for improved classification of AD/MCI patients. IEEE J Biomed Health Inform. (2015) 19:1610–6. 10.1109/JBHI.2015.2429556 25955998 PMC4573581

[B23] Chirles TJ Reiter K Weiss LR Alfini AJ Nielson KA Smith JC. Exercise training and functional connectivity changes in mild cognitive impairment and healthy elders. J Alzheimers Dis. (2017) 57:845–56. 10.3233/JAD-161151 28304298 PMC6472271

[B24] Risacher SL Tallman EF West JD Yoder KK Hutchins GD Fletcher JW Olfactory identification in subjective cognitive decline and mild cognitive impairment: Association with tau but not amyloid positron emission tomography. Alzheimers Dement Diagn Assess Dis Monitor. (2017) 9:57–66. 10.1016/j.dadm.2017.09.001 PMC5675709 29159268

[B25] Wang Z Jia X Liang P Qi Z Yang Y Zhou W. Changes in thalamus connectivity in mild cognitive impairment: evidence from resting state fMRI. Eur J Radiol. (2012) 81:277–85. 10.1016/j.ejrad.2010.12.044 21273022

[B26] Liu M Zhang D Shen D. Relationship induced multi-template learning for diagnosis of Alzheimer's disease and mild cognitive impairment. IEEE Trans Med Imag. (2016) 35:1463–74. 10.1109/TMI.2016.2515021 26742127 PMC5572669

[B27] Wang B Niu Y Miao L Cao R Yan P Guo H. Decreased complexity in Alzheimer's disease: resting-state fMRI evidence of brain entropy mapping. Front Aging Neurosci. (2017) 9:378. 10.3389/fnagi.2017.00378 29209199 PMC5701971

[B28] Jin C Chao Y-P Lin L Fu Z Zhang B Wu S The study of graph measurements for hub identification in multiple parcellated brain networks of healthy older adult. J Med Biol Eng. (2017) 37:653–65. 10.1007/s40846-017-0259-8

[B29] Bujnoskova E Marecek R Mikl M Fousek J Selnes P Hessen E ID 326-Functional connectivity alterations and their relation to pathophysiological changes in mild cognitive impairment. Clin Neurophysiol. (2016) 127:e126–7. 10.1016/j.clinph.2015.11.429

[B30] Pereira JB Mijalkov M Kakaei E Mecocci P Vellas B Tsolaki M Abnormal network organization in patients with mild cognitive impairment and Alzheimer's disease. Alzheimers Dement (2016) 12:P34 10.1016/j.jalz.2016.06.048 PMC4961019 27178195

[B31] Mathotaarachchi SS Pascoal TA Shin M Benedet AL Kang M-S Struyfs H Graph-theory analysis shows a highly efficient but redundant network in mci tau propagation. Alzheimers Dement. (2017) 13:P1275–6. 10.1016/j.jalz.2017.06.1914

[B32] Song S Zhan Z Long Z Zhang J Yao L. Comparative study of SVM methods combined with voxel selection for object category classification on fMRI data. PLoS ONE (2011) 6:e17191. 10.1371/journal.pone.0017191 21359184 PMC3040226

[B33] Bi X-A Shu Q Sun Q Xu Q. Random support vector machine cluster analysis of resting-state fMRI in Alzheimer's disease. PLoS ONE (2018) 13:e0194479. 10.1371/journal.pone.0194479 29570705 PMC5865739

[B34] Zhang D Wang Y Zhou L Yuan H Shen D. Multimodal classification of Alzheimer's disease and mild cognitive impairment. NeuroImage (2011) 55:856–67. 10.1016/j.neuroimage.2011.01.008 21236349 PMC3057360

[B35] Granziera C Daducci A Donati A Bonnier G Romascano D Roche A. A multi-contrast MRI study of microstructural brain damage in patients with mild cognitive impairment. NeuroImage Clin. (2015) 8:631–9. 10.1016/j.nicl.2015.06.003 26236628 PMC4511616

[B36] Ye T Zu C Jie B Shen D Zhang D. Discriminative multi-task feature selection for multi-modality classification of Alzheimer's disease. Brain Imag Behav. (2016) 10:739–49. 10.1007/s11682-015-9437-x 26311394 PMC4769696

[B37] Long Z Jing B Guo R Li B Cui F Wang T. A Brainnetome Atlas based mild cognitive impairment identification using hurst exponent. Front Aging Neurosci. (2018) 10:103. 10.3389/fnagi.2018.00103 29692721 PMC5902491

[B38] Destrieux C Terrier LM Andersson F Love SA Cottier J-P Duvernoy H. A practical guide for the identification of major sulcogyral structures of the human cortex. Brain Struct Func. (2017) 222:2001–15. 10.1007/s00429-016-1320-z 27709299

[B39] Fang S Wang Y Jiang T. The influence of frontal lobe tumors and surgical treatment on advanced cognitive functions. World Neurosurg. (2016) 91:340–6. 10.1016/j.wneu.2016.04.006 27072331

[B40] Joo MS Park DS Moon CT Chun YI Song SW Roh HG. Relationship between gyrus rectus resection and cognitive impairment after surgery for ruptured anterior communicating artery aneurysms. J Cerebrovasc Endovasc Neurosurg. (2016) 18:223–8. 10.7461/jcen.2016.18.3.223 27847765 PMC5104846

[B41] Qiu A Mori S Miller MI. Diffusion tensor imaging for understanding brain development in early life. Ann Rev Psychol. (2015) 66:853–76. 10.1146/annurev-psych-010814-015340 25559117 PMC4474038

[B42] Kristine MK Olga Dal M Selene S Eric MW Vanessa R Jordan G Areas of brain damage underlying increased reports of behavioral disinhibition. J Neuropsychiatry Clin Neurosci. (2015) 27:193–8. 10.1176/appi.neuropsych.14060126 25959040 PMC6126363

[B43] Georgiopoulos C Warntjes M Dizdar N Zachrisson H Engstrom M Haller S. Olfactory impairment in Parkinson's disease studied with diffusion tensor and magnetization transfer imaging. J Parkinsons Dis. (2017) 7:301–11. 10.3233/JPD-161060 28482644 PMC5438470

[B44] Frings L Yew B Flanagan E Lam BYK Hüll M Huppertz H-J Longitudinal gray and white matter changes in frontotemporal dementia and Alzheimer's disease. PLoS ONE (2014) 9:e90814 10.1371/journal.pone.0090814 24595028 PMC3940927

[B45] Xie Y Cui Z Zhang Z Sun Y Sheng C Li K. Identification of amnestic mild cognitive impairment using multi-modal brain features: a combined structural MRI and diffusion tensor imaging study. J Alzheimers Dis. (2015) 47:509–22. 10.3233/JAD-150184 26401572

[B46] Carr VA Bernstein JD Favila SE Rutt BK Kerchner GA Wagner AD. Individual differences in associative memory among older adults explained by hippocampal subfield structure and function. Proc Natl Acad Sci USA. (2017) 114:12075. 10.1073/pnas.1713308114 29078387 PMC5692588

[B47] Lao Y Nguyen B Tsao S Gajawelli N Law M Chui H. A T1 and DTI fused 3D corpus callosum analysis in MCI subjects with high and low cardiovascular risk profile. NeuroImage Clin. (2017) 14:298–307. 10.1016/j.nicl.2016.12.027 28210541 PMC5299209

[B48] Bahar-Fuchs A Chetelat G Villemagne VL Moss S Pike K Masters CL. Olfactory deficits and amyloid-beta burden in Alzheimer's disease, mild cognitive impairment, and healthy aging: a PiB PET study. J Alzheimers Dis. (2010) 22:1081–7. 10.3233/JAD-2010-100696 20930316

[B49] You Y Novak L Clancy K Li W Human olfactory cortex contributes to emotional and perceptual aspects of aversive associative learning and memory. bioRxiv (2017). 10.1101/193748

[B50] Yaniv C David P Wilson DA Dynamic cortical lateralization during olfactory discrimination learning. J Physiol. (2015) 593:1701–14. 10.1113/jphysiol.2014.288381 25604039 PMC4386967

[B51] Daniels JK Vermetten E. Odor-induced recall of emotional memories in PTSD–review and new paradigm for research. Exp Neurol. (2016) 284:168–80. 10.1016/j.expneurol.2016.08.001 27511295

[B52] Stone SSD Teixeira CM DeVito LM Zaslavsky K Josselyn SA Lozano AM. Stimulation of entorhinal cortex promotes adult neurogenesis and facilitates spatial memory. J Neurosci. (2011) 31:13469. 10.1523/JNEUROSCI.3100-11.2011 21940440 PMC6623309

[B53] Goto M Abe O Miyati T Yoshikawa T Hayashi N Takao H. Entorhinal cortex volume measured with 3T MRI is positively correlated with the Wechsler memory scale-revised logical/verbal memory score for healthy subjects. Neuroradiology (2011) 53:617–22. 10.1007/s00234-011-0863-1 21455719

[B54] Zhang B Zhang X Zhang F Li M Schwarz CG Zhang J. Characterizing topological patterns in amnestic mild cognitive impairment by quantitative water diffusivity. J Alzheimers Dis. (2015) 43:687–97. 10.3233/JAD-140882 25114082

[B55] Kirova A-M Bays RB Lagalwar S Working memory and executive function decline across normal aging, mild cognitive impairment, and Alzheimer's diease. BioMed Res Int. (2015) 2015:9 10.1155/2015/748212 PMC4624908 26550575

[B56] Vasavada MM Wang J Eslinger PJ Gill DJ Sun X Karunanayaka P. Olfactory cortex degeneration in Alzheimer's disease and mild cognitive impairment. J Alzheimers Dis. (2015) 45:947–58. 10.3233/JAD-141947 25633674

[B57] Guzman VA Carmichael OT Schwarz C Tosto G Zimmerman ME Brickman AM. White matter hyperintensities and amyloid are independently associated with entorhinal cortex volume among individuals with mild cognitive impairment. Alzheimers Dement J Alzheimers Assoc. (2013) 9:S124–31. 10.1016/j.jalz.2012.11.009 23375566 PMC3663926

[B58] Sakurai Y Furukawa E Kurihara M Sugimoto I. Frontal phonological agraphia and acalculia with impaired verbal short-term memory due to left inferior precentral gyrus lesion. Case Rep Neurol. (2018) 10:72–82. 10.1159/000487849 29681826 PMC5903121

[B59] Sakreida K Scorolli C Menz M Heim S Borghi A Binkofski F. Are abstract action words embodied? An fMRI investigation at the interface between language and motor cognition. Front Hum Neurosci. (2013) 7:125. 10.3389/fnhum.2013.00125 23576972 PMC3620530

[B60] Chang Y. Reorganization and plastic changes of the human brain associated with skill learning and expertise. Front Hum Neurosci. (2014) 8:35. 10.3389/fnhum.2014.00035 24550812 PMC3912552

[B61] Han SD Arfanakis K Fleischman DA Leurgans SE Tuminello ER Edmonds EC. Functional connectivity variations in mild cognitive impairment: associations with cognitive function. J Int Neuropsychol Soc. (2011) 18:39–48. 10.1017/S1355617711001299 22005016 PMC3368801

[B62] Rose SE McMahon KL Janke AL O'Dowd B de Zubicaray G Strudwick MW. Diffusion indices on magnetic resonance imaging and neuropsychological performance in amnestic mild cognitive impairment. J Neurol Neurosurg Psychiatry (2006) 77:1122–8. 10.1136/jnnp.2005.074336 16754694 PMC2077533

[B63] Lin F Ren P Lo RY Chapman BP Jacobs A Baran TM. Insula and inferior frontal gyrus' activities protect memory performance against Alzheimer's disease pathology in old age. J Alzheimers Dis. (2017) 55:669–78. 10.3233/JAD-160715 27716674 PMC5531269

[B64] Mickley Steinmetz KR Addis DR Kensinger EA. The effect of arousal on the emotional memory network depends on valence. NeuroImage (2010) 53:318–24. 10.1016/j.neuroimage.2010.06.015 20542121 PMC3063443

[B65] van Dam WO Decker SL Durbin JS Vendemia JMC Desai RH. Resting state signatures of domain and demand-specific working memory performance. NeuroImage (2015) 118:174–182. 10.1016/j.neuroimage.2015.05.017 25980975 PMC4554837

[B66] Lauer J Moreno-López L Manktelow A Carroll EL Outtrim JG Coles JP. Neural correlates of visual memory in patients with diffuse axonal injury. Brain Inj. (2017) 31:1513–20. 10.1080/02699052.2017.1341998 28707953

[B67] Arsalidou M Morris D Taylor MJ. Converging evidence for the advantage of dynamic facial expressions. Brain Topogr. (2011) 24:149–63. 10.1007/s10548-011-0171-4 21350872

[B68] Jacobs HIL Van Boxtel MPJ Heinecke A Gronenschild EHBM Backes WH Ramakers IHGB. Functional integration of parietal lobe activity in early Alzheimer disease. Neurology (2012) 78:352. 10.1212/WNL.0b013e318245287d 22262753

[B69] Alexopoulos P Sorg C Förschler A Grimmer T Skokou M Wohlschläger A. Perfusion abnormalities in mild cognitive impairment and mild dementia in Alzheimer's disease measured by pulsed arterial spin labeling MRI. Eur Arch Psychiatry Clin Neurosci. (2012) 262:69–77. 10.1007/s00406-011-0226-2 21786091

[B70] Makizako H Shimada H Doi T Park H Yoshida D Suzuki T. Six-minute walking distance correlated with memory and brain volume in older adults with mild cognitive impairment: a voxel-based morphometry study. Dement Geriatr Cogn Dis Extra (2013) 3:223–32. 10.1159/000354189 24052797 PMC3776400

